# Clinically useful limited sampling strategy to estimate area under the concentration-time curve of once-daily tacrolimus in adult Japanese kidney transplant recipients

**DOI:** 10.1371/journal.pone.0225878

**Published:** 2019-12-11

**Authors:** Ryuto Nakazawa, Miki Yoshiike, Shiari Nozawa, Koichiro Aida, Yuichi Katsuoka, Eisuke Fujimoto, Masahiko Yazawa, Eiji Kikuchi, Yugo Shibagaki, Hideo Sasaki

**Affiliations:** 1 Department of Urology, St. Marianna University School of Medicine, Kawasaki, Japan; 2 Division of Nephrology and Hypertension, Department of Internal Medicine, St. Marianna University School of Medicine, Kawasaki, Japan; Medical University of Gdansk, POLAND

## Abstract

**Background:**

An extended-release, once-daily, oral formulation of tacrolimus is currently used after kidney transplantation as a substitute for the conventional twice-daily formulation. The purpose of this study was to provide a limited sampling strategy with minimum and optimum sampling points to predict the tacrolimus area under the concentration-time curve (AUC) after administration of once-daily tacrolimus in *de novo* adult kidney transplant patients.

**Methods:**

A total of 36 adult Japanese kidney transplant patients receiving once-daily tacrolimus were included: 31 were allocated to a study group to develop limited sampling strategy (LSS) model equations based on multiple stepwise linear regression analysis, and 5 were allocated to a validation group to estimate the precision of the LSS equations developed by the study group. Twelve-hour AUC (AUC_0-12_) was calculated by the trapezoidal rule, and the relationship between individual concentration points and AUC_0-12_ were determined by multiple linear regression analysis. The coefficient of determination (R^2^) was used to assess the goodness-of-fit of the regression models. Three error indices (mean error, mean absolute error, and root mean squared prediction error) were calculated to evaluate predictive bias, accuracy, and precision, respectively. Quality of the statistical models was compared with Akaike's information criterion (AIC).

**Results:**

A four-point model using C_0_, C_2_, C_4_ and C_6_ gave the best fit to predict AUC_0-12_ (R^2^ = 0.978). In the three- and two-point models, the best fits were at time points C_2_, C_4_, and C_6_ (R^2^ = 0.973), and C_2_ and C_6_ (R^2^ = 0.962), respectively. All three models reliably estimated tacrolimus AUC_0-12_, consistent with evaluations by the three error indices and Akaike’s information criterion. Practically, the two-point model with C_2_ and C_6_ was considered to be the best combination, providing a highly accurate prediction and the lowest blood sampling frequency.

**Conclusions:**

The two-point model with C_2_ and C_6_ may be valuable in reducing the burden on patients, as well as medical costs, for once-daily tacrolimus monitoring.

## Introduction

Tacrolimus is an immunosuppressant with potent immunosuppressive properties that is mainly used after many types of solid organ transplantation. It suppresses the production of IL-2 derived from activated T-cells [1]. Tacrolimus is a drug with a narrow therapeutic window; the range of efficacy is close to that associated with toxicity. There is also a large inter-individual variation in the blood level of the drug after administration, even with the same dose based on body weight [2, 3]. For these reasons, therapeutic drug monitoring (TDM) is necessary to determine the appropriate dose for each patient, and the area under the curve (AUC) is a particularly useful parameter to determine the optimal dosing regimen due to its inter-individual consistency and ability to estimate individual profiles of the drug’s pharmacokinetics [4].

Until recently, tacrolimus was generally used as a twice-daily oral formulation at a dosage often determined based on trough blood concentration (C_0_) as a simplified marker of drug exposure, and this was reported to correlate well with the AUC [4, 5]. Alternatively, a limited sampling strategy (LSS) to reduce the frequency of blood sampling has been used to predict AUC [6]. Accurate AUC measurements require frequent blood sampling over a long time period after dosing.

In recent years, an extended-release formulation of tacrolimus has been used for recipients soon after kidney transplantation, allowing once-daily dosing that is expected to improve medication adherence and patient quality of life [7, 8]. Once-daily tacrolimus has been demonstrated to have similar efficacy and tolerability to conventional twice-daily tacrolimus [9], and has become a valuable alternative to twice-daily administration with appropriate dosage modifications [10, 11].

At present there are only a few reports on LSS to predict actual AUC after administration of once-daily tacrolimus, and several LSS models for once-daily tacrolimus in adult kidney transplant recipients have been proposed [12–15]. Most of such work has focused on the strong correlation between C_0_ and AUC_0-24_ [13–15]. The trough concentration has been reported to be important point for the tacrolimus LSS at which C_0_ plus any other single [15] or two point(s) [12–14] are recommended to estimate the AUC_0-24_. However, the optimal collection time and frequency of blood sampling for LSS are not conclusive.

The purpose of the present study was to determine the optimal sampling timing for daily clinical use and the minimum sampling frequency to estimate tacrolimus exposure after administration of once-daily tacrolimus in *de novo* kidney transplant recipients.

## Materials and methods

### Patients and study design

This study was approved by the Institutional Ethics Committee of St. Marianna University School of Medicine (approval number: 4032) as a retrospective observational study with medical information that required no informed consent. Among 44 adult Japanese patients who underwent renal transplantation at St. Marianna University School of Medicine hospital between January 2009 and August 2011, 36 received prolonged-release once-daily tacrolimus (Graceptor^®^, Astellas Pharma Inc, Japan: trade name Advagraf^®^ in Europe, Astagraf XL in the US) were included in this study. The 31 patients from January 2009 to May 2011 were allocated to a study group to develop LSS model equations based on multiple stepwise linear regression analysis. The 5 patients from June to August 2011 were allocated to a validation group to estimate the precision of the LSS equations developed by the study group.

The opportunities of blood samplings to monitor tacrolimus concentrations were provided four times during the hospital stay after transplant operations (1 and 3 weeks after transplantation) and on protocol biopsies of stable transplants (8 weeks and 12 months after transplantation), as a routine practice for renal transplant patients covered by insurance in Japan. However not all patients made best use of these opportunities. Whole blood was withdrawn just before and 0.5, 2, 4, 6, 8 and 12 hours after oral administration of once-daily tacrolimus at 7:30. In a subset of the patients, whole blood was additively withdrawn 24 hours post-dose. Patients were allowed breakfast 30 minutes after medication. The initial dose at the time of transplantation was 0.15 mg/kg/day, and subsequent doses were titrated based on tacrolimus trough levels while monitoring the patient’s biochemistry parameters and clinical outcome, which were adjusted to 7–10 ng/ml in the first 1–3 postoperative week(s), and 5–7 ng/ml after the first 8 postoperative weeks. The levels of tacrolimus in whole blood samples were determined by the central laboratory in St. Marianna University School of Medicine Hospital using a chemiluminescent microparticle immunoassay system (ARCHITECT Tacrolimus, Abbott Laboratories, IL, USA) on an ARCHITECT i2000 autoanalyzer (Abbott) with in-house validation. The quantification range was 0.5–30 ng/ml.

### Pharmacokinetic evaluation and statistical analysis

The measured AUC_0-12_ was calculated by the linear trapezoidal rule using the data on tacrolimus blood levels 0, 0.5, 2, 4, 6, 8 and 12 hours after oral administration of once-daily tacrolimus. Tacrolimus concentrations at each sampling time were calculated by linear regression analysis with the measured tacrolimus AUC_0-12_. Tacrolimus concentrations at sampling time points that showed the best correlations were combined by multiple stepwise linear regression analysis to give improved correlations with measured AUC_0-12_. R^2^ (coefficient of determination) was used to assess the goodness-of-fit of the regression models.

Three prediction error indices, mean error (ME), mean absolute error (MAE), and root mean squared prediction error (RMSE), were calculated with the following equations to evaluate predictive bias, accuracy, and precision, respectively. The clinically acceptable percentage limits of ME, MAE, and RMAE were, 5%, 10%, and 15%, respectively [12]. In Eqs (1)–(3), “pred” was the predicted value of AUC, “mes” was the measured value of AUC determined with 7 time points (0, 0.5, 2, 4, 6, 8 and 12 hours), and “N” was the number of patients.

$$ME\;(\%)=\frac{1}{N}\sum_{i=1}^{N}\left(\frac{pred-mes}{pred}\right)\times 100$$ (1)

$$MAE\;(\%)=\frac{1}{N}\sum_{i=1}^{N}\left|\frac{pred-mes}{pred}\right|\times 100$$ (2)

$$RMSE\;(\%)=\sqrt{\frac{1}{N}\sum_{i=1}^{N}{\left(\frac{pred-mes}{pred}\right)}^{2}}\times 100$$ (3)

In order to compare the prediction models, Akaike’s information criterion (AIC) [16], shown in Eq (4), was used. AIC can be used to compare different model structures based on the number of parameters (Nparams), and an additional metric to determine the ability of the respective models to predict the data. A lower AIC is indicative of a superior prediction model. In Eq (4), “RSS” was the residual sum of squares, used as the metric for goodness-of-fit, and “Npts” was the number of data points.

$$AIC=Npts\;ln\;\left(\frac{RSS}{Npts}\right)+2\;Nparams$$ (4)

Bland-Altman analysis and Pearson’s correlation coefficient test were applied to assess the agreement and correlation between the measured AUC and predicted AUC, respectively. Statistical analysis was performed with JMP ver 13 (SAS Institute, Cary, NC).

## Results

During the study period, 98 tacrolimus pharmacokinetics profiles from 0 to 12 hours post-dose were analyzed from 36 patients (68.1%; 98/36x4) in total: 79 from 31 for the study group (57.3%; 79/31x4) and 17 from 5 for the validation group (85.0%, 17/5x4), with which we conducted the present analyses retrospectively (S1 Fig). Baseline characteristics of all the patients (total, study group, and validation group) included in this study are summarized in Table 1. Before completion of the study, two patients withdrew from medication due to hyperkalemia and rising serum creatinine, two patients changed drug because of acute rejection, and one patient died from postoperative infective endocarditis.

**Table 1:** Demographics and characteristics of the patients in this study.

Parameters	Total	Study group	Validation group
Patients, n	36	31	5
Age (years)	45.2 ± 14.9	44.8 ± 14.9	49.8 ± 15.3
Male/female, n	21/15	18/13	3/2
Weight (kg)	55.0 ± 14.5	55.6 ± 11.3	49.9 ± 26.2
BMI (kg/m^2^)	20.8 ± 14.5	21.0 ± 3.0	19.4± 9.1
ABO compatible/incompatible, n	29/7	27/4	2/3
Virus infection, n			
CMV	1	1	0
VZV	1	1	0
Acute rejection, n	2	2	0
Total AUC measurements, n	96	79	17
Serum creatinine (mg/dl)	1.25 ± 0.37	1.26 ± 0.33	1.20 ± 0.53
Serum K (mEq/l)	4.43 ± 0.52	4.47 ± 0.54	4.22 ± 0.40
Hemoglobin (g/dl)^a^	11.0 ± 1.77	10.9 ± 1.76	11.3 ± 182
Hematocrit (%)^b^	33.5 ± 5.26	33.2 ± 5.21	34.6 ± 5.52
Dose of tacrolimus (mg/kg)	0.113 ± 0.041	0.116 ± 0.039	0.101 ± 0.052
Trough of tacrolimus (ng/ml)	6.61 ± 3.01	6.46 ± 2.67	7.33 ± 4.30
AUC (ng/ml)	162.9 ± 52.8	162.7 ± 50.9	163.7 ± 61.7

number or mean ± SD. SD, standard deviation; BMI, body mass index; CMV, cytomegalovirus; VZV, varicella zoster virus; AUC, area under the curve.

^a^n = 78.

^b^n = 77.

**Fig 1:**
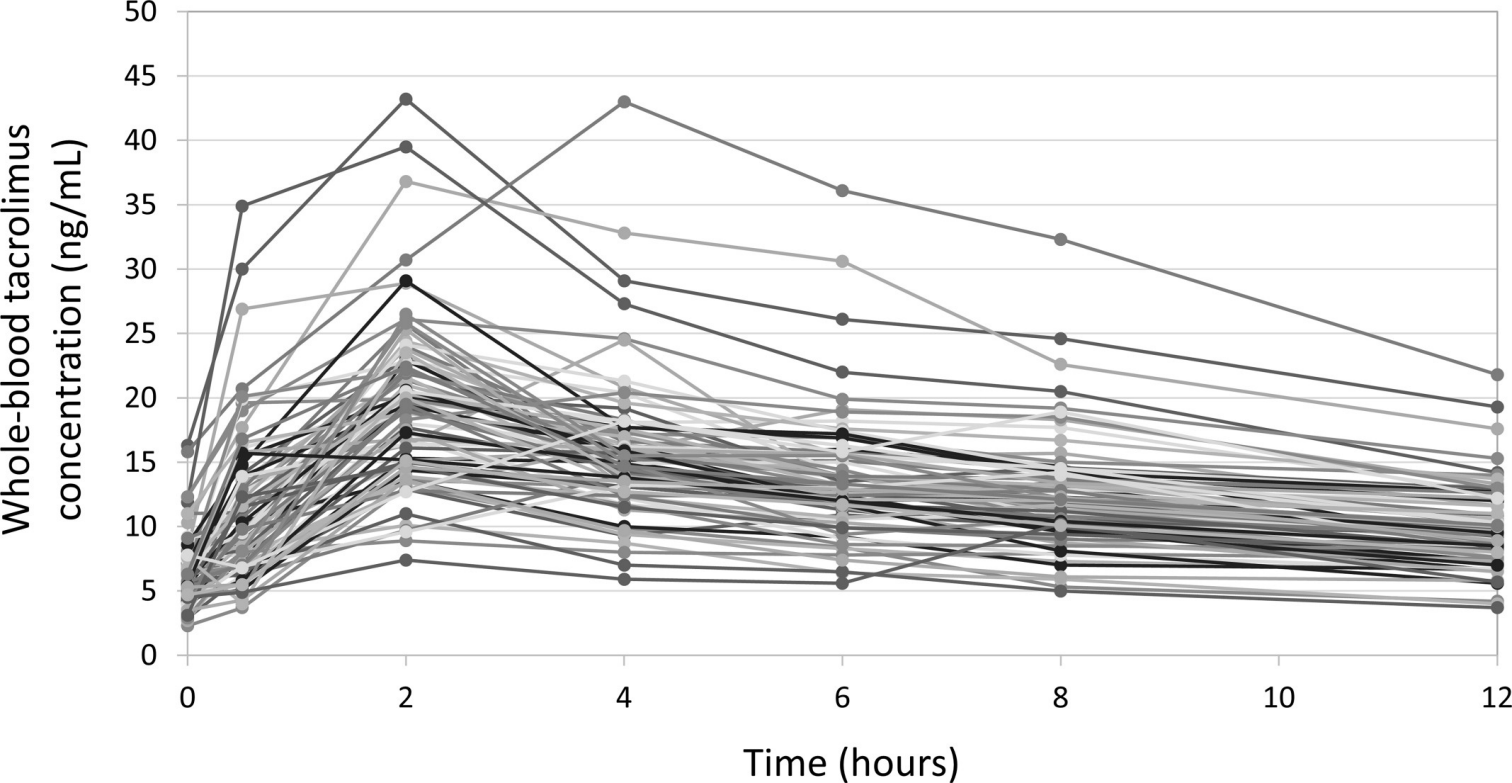
A total of 79 tacrolimus concentration-time curves completed with data from the 31 study group patients. The x-axis represents time points after oral administration of once-daily tacrolimus. The y-axis represents the whole-blood tacrolimus level. On most of the curves, blood tacrolimus concentrations rose to maximum levels (C_max_) at 2 hours post-administration and gradually decreased thereafter.

In the study group, the number of concentration-time curves available at 1 week, 3 weeks, 8 weeks, and 12 months after transplantation were 22, 22, 16, and 19, respectively. On most of the curves (70/79), blood tacrolimus concentrations rose to the maximum levels (C_max_) at 2 hours post-administration and gradually decreased thereafter (Fig 1). Only nine exceptions were noted; C_max_ at 0.5, 4, and 6 hours post-administration for 2, 6, and 1 patient(s), respectively.

**Fig 2:**
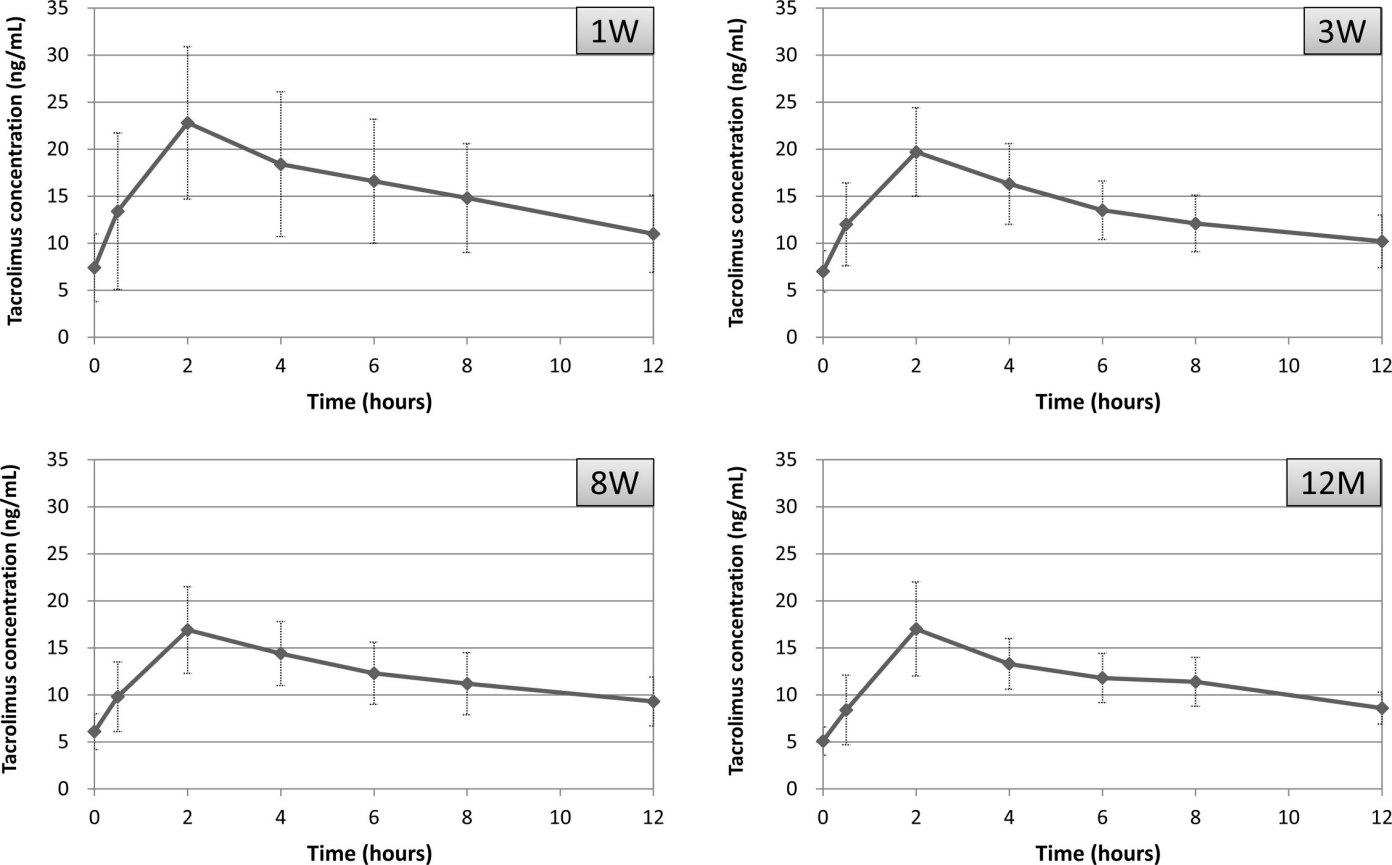
Concentration-time curves of the study group patients at post-operative 1 week, 3 weeks, 8 weeks and 12 months. The x-axis represents time points after oral administration of once-daily tacrolimus. The y-axis represents the whole-blood mean (SD) tacrolimus level. On most of the curves, blood tacrolimus concentrations rose to maximum levels (C_max_) at 2 hours post-administration and gradually decreased thereafter. Numbers of profiles at post-operative 1 week, 3 weeks, 8 weeks and 12 months were 22, 22, 16, and 19, respectively.

Baseline tacrolimus blood levels were variable according to the period after transplantation; however, tacrolimus concentration-time curves showed a very similar pattern at any post-operative period, i.e., at 1 week, 3 weeks, 8 weeks and 12 months (Fig 2).

Predictive performances of the limited sampling equations with various sampling time points are shown in Table 2. In equations with a single time point concentration, the R^2^ value was the lowest at C_0_ (0.673), increasing thereafter to reach a maximum at C_6_ (0.914), and decreasing from then on. The authors accordingly decided to perform stepwise multiple regression analyses using time points before C_6_. The four-point equation using C_0_, C_2_, C_4_ and C_6_ was the best fit to predict AUC_0-12_ (R^2^ = 0.978). In the three- and two-point equations, the best fits were time points at C_2_, C_4_, and C_6_ (R^2^ = 0.973), and C_2_ and C_6_ (R^2^ = 0.962), respectively. On the whole, combinations with C_6_ *without* C_0_ were found to be favorable. The three error indices for all model equations were below the clinically acceptable percentage limits, except for those with a single point C_0_ and C_2_, at which MAE and RMSE exceeded the percentage limit.

**Table 2:** Predictive performances of the limited sampling equations for AUC with various sampling time points.

Sampling point		R^2^	Model equations for AUC	Prediction error (%)			AIC
				ME	MAE	RMSE	
One	C_0_	0.673	C_0_x15.704+61.203	-3.169	14.531	18.113	536.5
	C_2_	0.692	C_2_x6.626+34.433	-2.410	12.618	17.560	531.6
	C_4_	0.896	C_4_x8.838+23.165	-1.070	7.633	10.226	446.2
	C_6_	0.914	C_6_x10.364+20.787	-0.881	6.888	9.268	430.7
	C_8_	0.875	C_8_x11.225+22.443	-1.110	8.026	11.185	460.4
	C_12_	0.848	C_12_x15.053+14.557	-1.002	9.714	12.345	476.0
Four	C_0_, C_2_, C_4_, C_6_	0.978	C_0_x2.232+C_2_x1.998+C_4_x2.539+C_6_x4.718+4.890	-0.274	4.064	4.784	329.1
Three	C_2_, C_4_, C_6_	0.973	C_2_x2.136+C_4_x2.699+C_6_x5.397+4.827	-0.277	4.293	5.277	343.6
	C_0_, C_2_, C_6_	0.969	C_0_x2.494+C_2_x2.292+C_6_x7.051+5.642	-0.334	4.563	5.691	355.5
	C_0_, C_2_, C_4_	0.954	C_0_x3.715+C_2_x2.127+C_4_x5.654+8.251	-0.501	5.700	6.849	384.8
	C_0_, C_4_, C_6_	0.950	C_0_x3.715+C_2_x2.127+C_4_x5.654+8.251	-0.501	5.700	7.187	392.4
Two	C_2_, C_6_	0.962	C_2_x2.468+C_6_x7.981+5.623	-0.342	4.926	6.204	368.2
	C_4_, C_6_	0.940	C_4_x3.981+C_6_x6.079+16.603	-0.678	5.633	7.820	404.8
	C_2_, C_4_	0.939	C_2_x2.415+C_4_x6.773+9.014	-0.567	6.649	7.887	406.2
	C_0_, C_6_	0.929	C_0_x3.677+C_6_x8.743+19.219	-0.813	6.133	8.503	418.0
	C_0_, C_4_	0.922	C_0_x4.747+C_4_x7.094+20.024	-0.909	6.796	8.885	425.0
	C_0_, C_2_	0.834	C_0_x9.352+C_2_x4.150+21.921	-1.178	9.801	12.972	484.8

AUC, area under the curve; ME, mean error; MAE, mean absolute error; RMSE, root mean squared error; AIC, Akaike’s information criterion.

**Fig 3:**
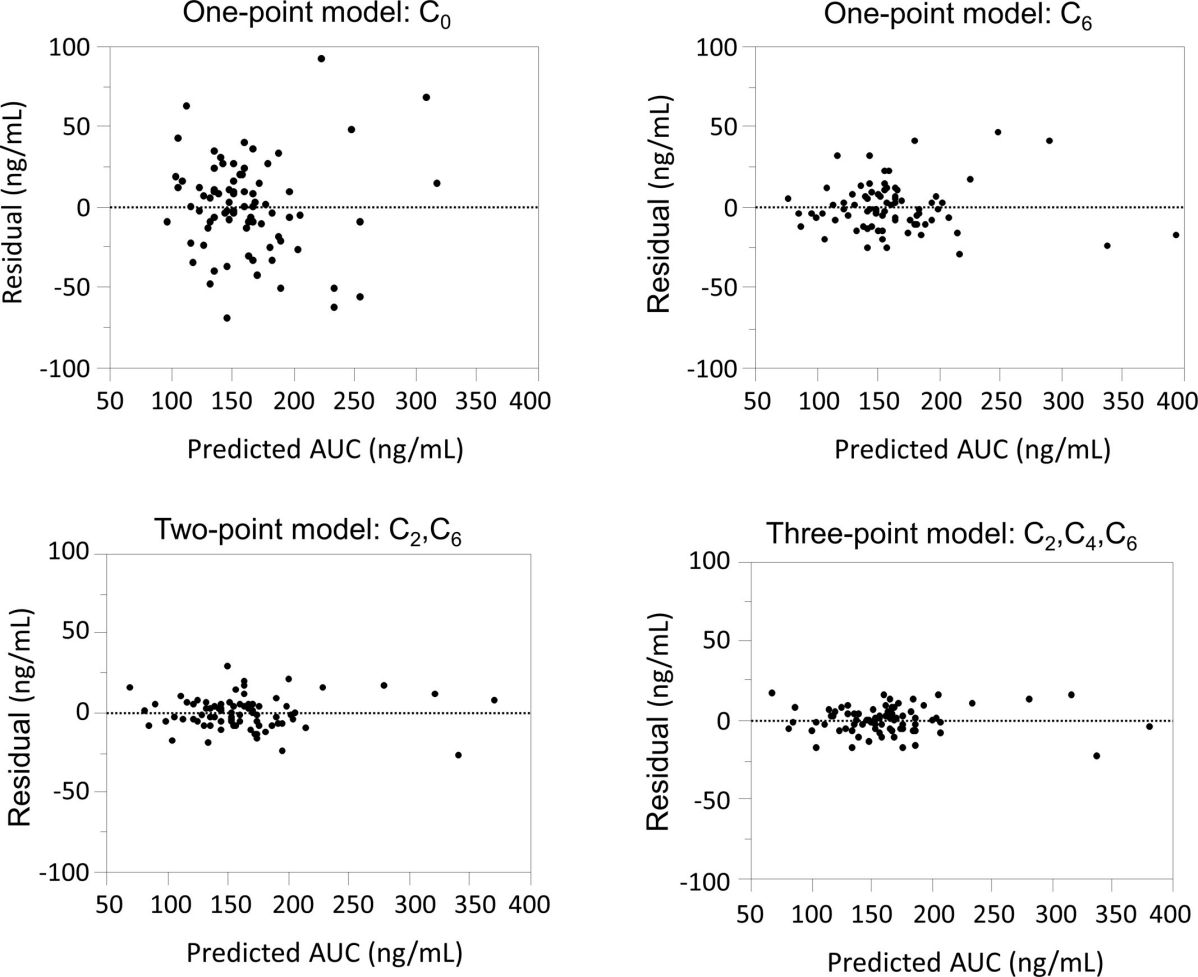
Bland-Altman plots of predictive AUC on the x-axis versus residuals (differences between AUC_0-12_ and predictive AUC) on the y-axis. The difference was largest for C_0_ and smallest for C_6_ for a one-point equation. The combinations of sampling points with C_6_ without C_0_ (C_2_ and C_6_, or C_2_, C_4_ and C_6_) were less variable.

A Bland-Altman plot of predictive AUC versus differentials between AUC_0-12_ and predictive AUC indicated that the difference was largest when using C_0_ and the smallest when using C_6_ for a one-point equation, and that the combinations of sampling points with C_6_ *without* C_0_ (C_2_ and C_6_, or C_2_, C_4_ and C_6_) were found to be less variable (Fig 3).

**Fig 4:**
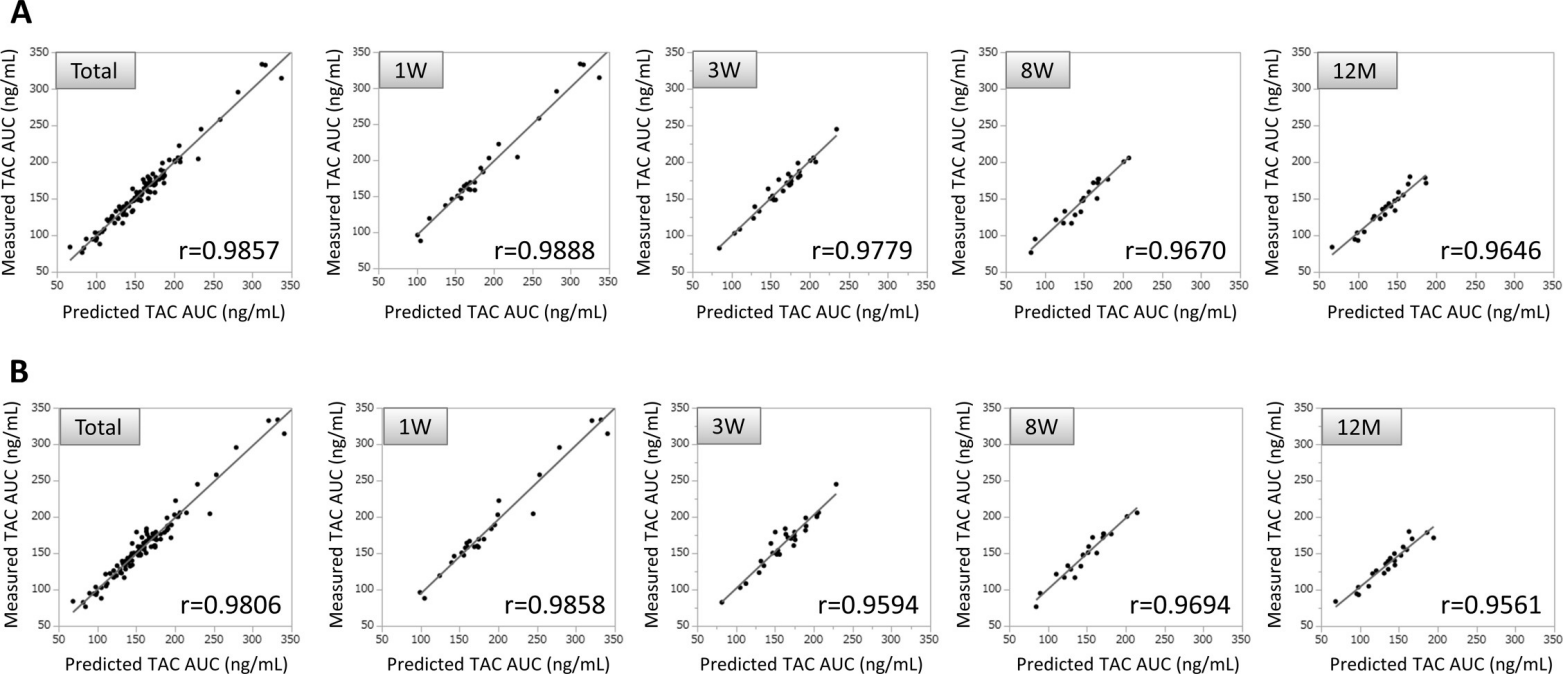
Scatter plots of predictive AUC on the x-axis versus measured AUC on the y-axis for the study group patients. The predicted AUC estimated with a multiple regression equation provided the closest approximation to the actual value of AUC_0-12_ using three time points with C_2_, C_4_, and C_6_ (A), and two time points with C_2_ and C_6_ (B). Very high correlations were observed from a total of 79 tacrolimus concentration-time curves, as well as from those observed separately at the post-operative period 1 week, 3 weeks, 8 weeks and 12 months (A, B).

Very high correlations between predictive AUC and the actual value of AUC_0-12_ were observed from a total of 79 tacrolimus concentration-time curves of the study group patients when using three time points with C_2_, C_4_, and C_6_ (r = 0.9857, Fig 4A) and two time points with C_2_ and C_6_ (r = 0.9806, Fig 4B). Comparable levels with a very high correlation were also found when observed separately at post-operative 1 week, 3 weeks, 8 weeks and 12 months (Fig 4A and 4B).

**Fig 5:**
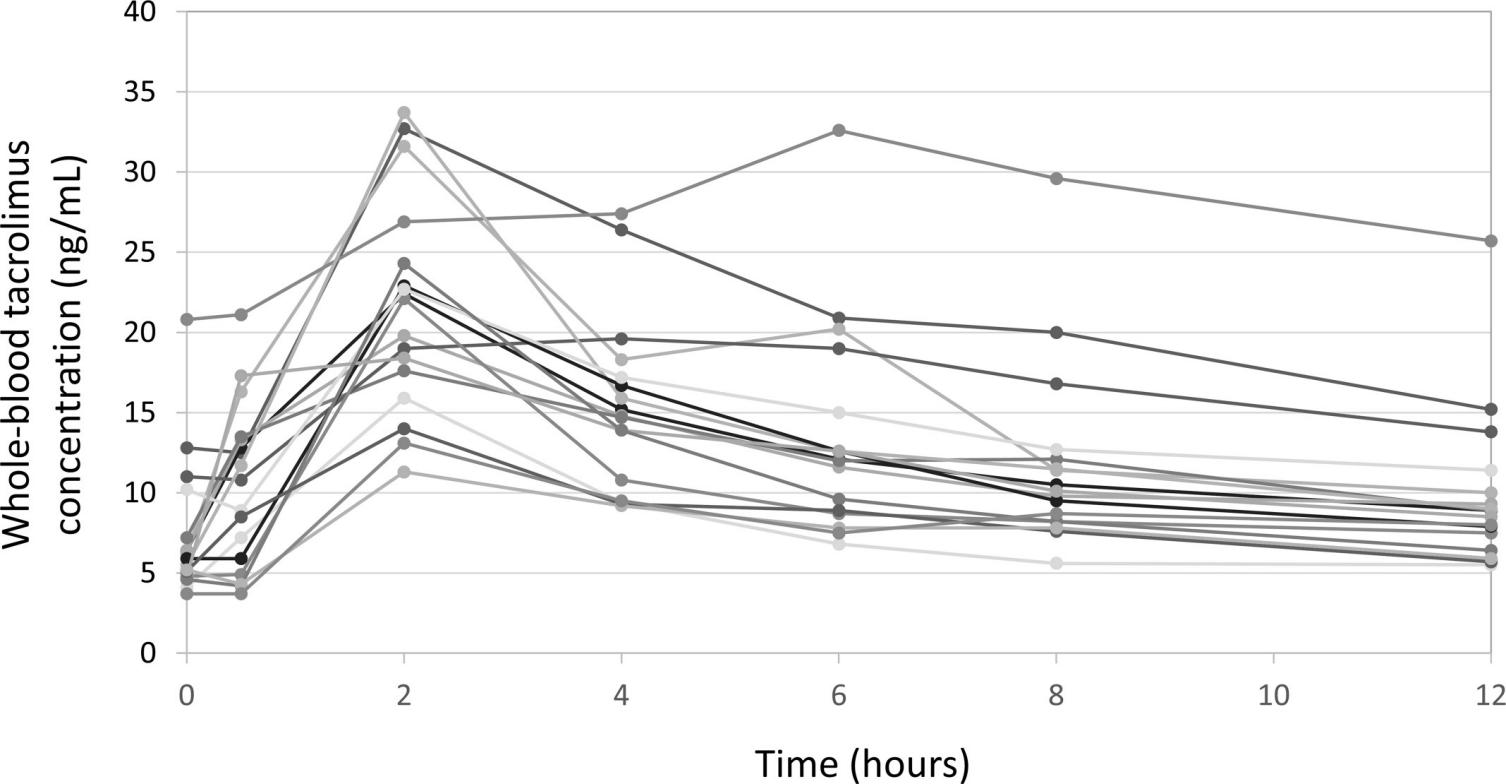
A total of 17 tacrolimus concentration-time curves completed with data from the 5 validation group patients. The x-axis represents time points after oral administration of once-daily tacrolimus. The y-axis represents the whole-blood tacrolimus level. Blood tacrolimus concentrations rose to C_max_ at 2 hours post-administration and gradually decreased thereafter.

Baseline characteristics of the patients in the independent validation group are summarized in Table 1. A total of 17 tacrolimus concentration-time curves were obtained from five patients. The number of curves available at 1 week, 3 weeks, 8 weeks, and 12 months after transplantation were 5, 5, 4, and 3, respectively. On most of the curves (15/17), blood tacrolimus concentrations rose to the maximum levels (C_max_) at 2 hours post-administration and gradually decreased thereafter (Fig 5), which was a very similar pattern to the study group (Fig 1).

**Fig 6:**
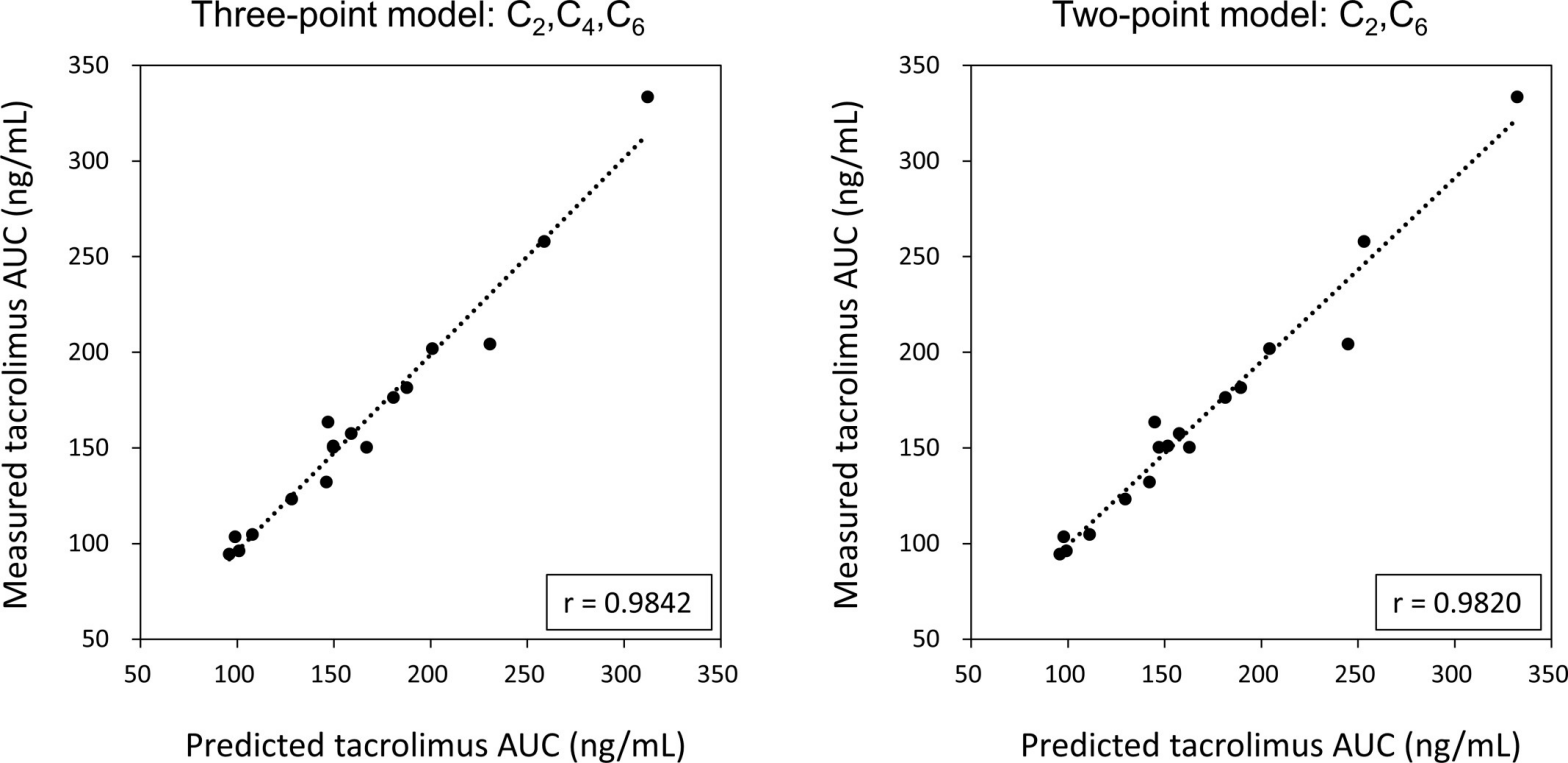
Scatter plots of predictive AUC on the x-axis versus measured AUC on the y-axis. The predicted AUC estimated with a multiple regression equation provided the closest approximation to the actual value of AUC_0-12_ using the three time points C_2_, C_4_, and C_6_, and almost exactly the same result was obtained using the two time points C_2_ and C_6_.

The predicted AUC of the validation group estimated with a multiple regression equation provided the closest approximation to the actually measured AUC_0-12_ at the three time points C_2_, C_4_, and C_6_ (r = 0.9842), and almost exactly the same result was obtained using the two time points C_2_ and C_6_ (r = 0.9820) (Fig 6).

In 13 patients from the study group (S2 Fig), correlations were analyzed between AUC_0-12_ and AUC_0-24_. The regression equation and squared correlation coefficient were AUC_0-24_ = 16.526+1.4887*AUC_0-12_ (r^2^ = 0.9571). (S3 Fig)

## Discussion

Once-daily tacrolimus was reported to have equivalent efficacy and safety to twice-daily tacrolimus [10, 17, 18]. The conversion of the tacrolimus formulation from twice-daily to once-daily can be safely implemented without sacrificing immunosuppressive effects [9, 11]. This conversion, however, requires careful attention because the pharmacokinetics of the once-daily formulation of tacrolimus is not identical to those of the twice-daily one. Furthermore, due to a large inter-individual variation in tacrolimus kinetics in the blood, even a small dosage of the drug may result in adverse events such as nephrotoxic nephritis and diabetes mellitus [19]. AUC provides more accurate information than trough concentration for regulating dosage with reduced adverse effects. However, drug monitoring with AUC is inconvenient because frequent blood collection is required, and this places a considerable physical and cost burden on the patient. Therefore, the trough concentration tends to be used in different situations for dosage modification [20].

The most conspicuous difference between our findings and other studies is the importance of C_0_ in once-daily tacrolimus LSS. The trough concentration of once-daily tacrolimus was reported to highly correlate with its AUC [14–15, 20–22] and to be an important sampling point to predict AUC_0-24_ [13–15]. Although the present study clearly found a considerable correlation between trough concentrations (C_0_) and AUC_0-12_ (R^2^ = 0.673), a one-point equation using C_0_ gave less fit to predict AUC_0-12_, suggesting that the trough concentration alone is inappropriate to estimate predicted AUC, even though it is helpful as a guide for dose determination. Results from our study indicated that R^2^ value was the lowest in C_0_ (0.673) in the single time point equations, at which MAE (14.531%) and RMSA (18.113%) exceeded the clinically acceptable percentage limits (the same holds for C2; R^2^ = 0.692). This is a reason why we considered C_0_ to be eliminated. Regarding the three- and two-point equations, we concluded that combinations *without* C_0_ were favorable because R^2^ values were lowered in the presence of C_0_. Nevertheless, R^2^ values for the equations with C_0_ were nearly comparable to those without C_0_ except two-point model using C_0_ and C_2_ (R^2^ = 0.834). Judging from R^2^ itself, It could be argued that equations with C_0_ are available.

In the prediction formula for AUC using a single time point, C_12_ was reported to give the highest correlation with the observed AUC_0-24_ (r^2^ = 0.9057) [15]. In contrast, C_6_ showed the best fit in the present study (R^2^ = 0.914) and R^2^ values subsequently decreased. Equations using a single time point are less accurate and are not recommended. As expected, the four-point model equation with C_0_, C_2_, C_4_ and C_6_ was the most accurate, and the predicted value from the model was proximate to the observed value.

Three-point models were sufficiently accurate in every combination, and the best was using the time points C_2_, C_4_ and C_6_. In the two-point models, the accuracy of the prediction equation varied depending on which time points were used. Overall, the prediction equation using C_2_ and C_6_ resulted in the best prediction accuracy. This can be attributed to the absence of the time point with the smallest R^2^ (C_0_), and the presence of the time point with the largest R^2^ (C_6_), which held true for the three-point models.

Validation with the independent patient group proved the practicability of the three-point model using C_2_, C_4_ and C_6_ (C_2_x2.136+C_4_x2.699+C_6_x5.397+4.827) and the two-point model using C_2_ and C_6_ (C_2_x2.468+C_6_x7.981+5.623). The predictive values obtained from these models were almost equivalent to the actual measured values (Fig 6), confirming that both model equations were practical to predict tacrolimus AUC.

In regression analysis, the value of R^2^ increases with an increased number of sampling time points; however, the accuracy of the prediction formula does not practically improve if there are four or more time points. For maximum effect with minimum effort in clinical settings, especially for outpatients, the two-point model using C_2_ and C_6_ is recommended.

Surprisingly, our LSS models for once-daily tacrolimus were found to be feasible for patients at any post-transplant period; at 1 week, 2 weeks, 8 weeks and 12 months. Generally, tacrolimus blood levels are relatively high immediately after transplantation and settle down to lower level within one year. However, the tacrolimus concentration-time curves showed a very similar pattern irrespective of the timing of blood sampling after transplantation in this study (Fig 2); blood tacrolimus concentrations rose to the maximum levels (C_max_) at 2 hours post-administration and gradually decreased thereafter. This uniformity in the pharmacokinetic profiles may reflect very high correlations between the predictive and actual value of the AUC (Fig 4). In once-daily tacrolimus time-concentration curves, C_max_ have often been reported to be at or near 2 hours post-dose; however, most of them showed large inter-individual variations [6, 12–15, 21, 22]. Small variations of tacrolimus pharmacokinetic profiles in the present study could be involved in the precise time of dosing and blood collections that were able to realize by managing the patients in hospital.

The pharmacokinetics of tacrolimus are variable depending on different conditions such as individual differences in absorption from the gastrointestinal tract [23–25], and the presence of hepatic dysfunction, which provokes decreased drug metabolism [26, 27]. In such cases, it is difficult to predict the tacrolimus AUC based on the limited sampling method because time to C_max_ is often prolonged and the discrepancy between the calculated and measured AUC becomes prominent. It should be noted that an actual AUC estimation is prerequisite for patients with atypical tacrolimus pharmacokinetics.

The major limitation of this study is that AUC_0-12_ was used as a guide for tacrolimus exposure, while AUC_0-24_ was more appropriate for once-daily tacrolimus pharmacokinetics. As this study was based on a retrospective analysis of hospital records, the data from our routine blood sampling for renal transplant patients’ TDM at that time (0 to 12 hours post-dose) were required for analysis. Nevertheless, we consider our LSS for once-daily tacrolimus monitoring is valuable because very high correlations between AUC_0-12_ and AUC_0-24_ were confirmed by using the data from a subset of the study group patients (S2 Fig and S3 Fig), suggesting only a small impact on the predictive performance of our proposed pharmacokinetic models.

## Conclusions

This study found that C_6_ was the most important time point in our limited sampling model to estimate the AUC of once-daily tacrolimus, while C_0_ (trough level) was found to be less appropriate for this purpose. The best combination of time points was C_2_ and C_6_ and this provided a highly accurate prediction with the lowest frequency of blood sampling, which may help to reduce both the burden on patients and medical costs.

## Supporting information

S1 Fig Patient allocation flow chart. (PDF)

S2 Fig Tacrolimus concentration-time curves for the 13 patients from the study group. (PDF)

S3 Fig Scatter plot of AUC0-12 versus AUC0-24 in the 13 patients from the study group. (PDF)
