# Supplementary figures and images for: Clinically useful limited sampling strategy to estimate area under the concentration-time curve of once-daily tacrolimus in adult Japanese kidney transplant recipients
Source: PLoS One. 2019 Dec 11;14(12):e0225878. doi: 10.1371/journal.pone.0225878 (PMC6905578; doi:10.1371/journal.pone.0225878)

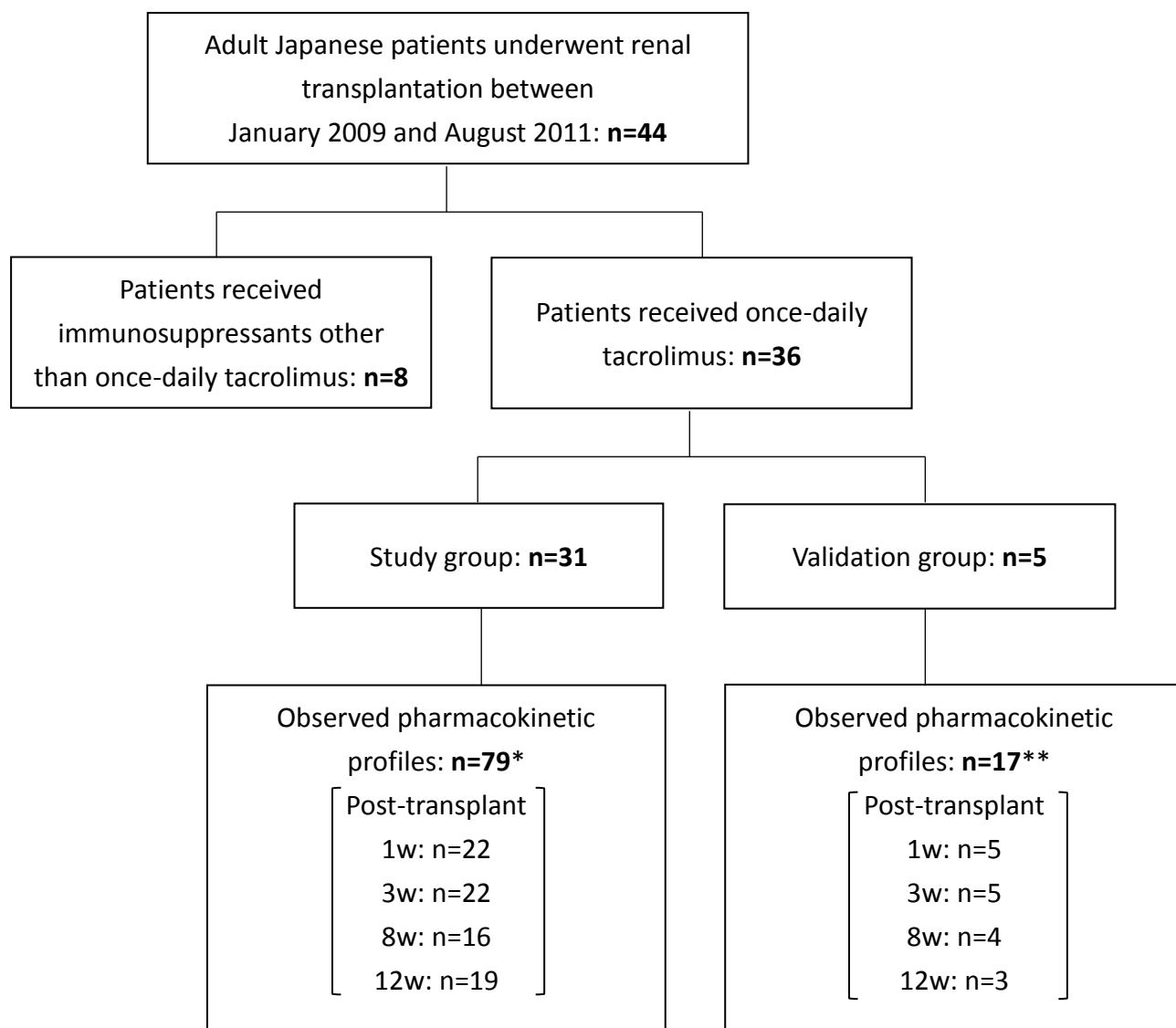

\*Of 124 (31x4), 79 (57.3%) pharmacokinetic profiles were available.

\*\*Of 20 (5x4), 17 (85.0%) pharmacokinetic profiles were available.

Supplement: S1 Fig — (PDF) [file pone.0225878.s001.pdf]

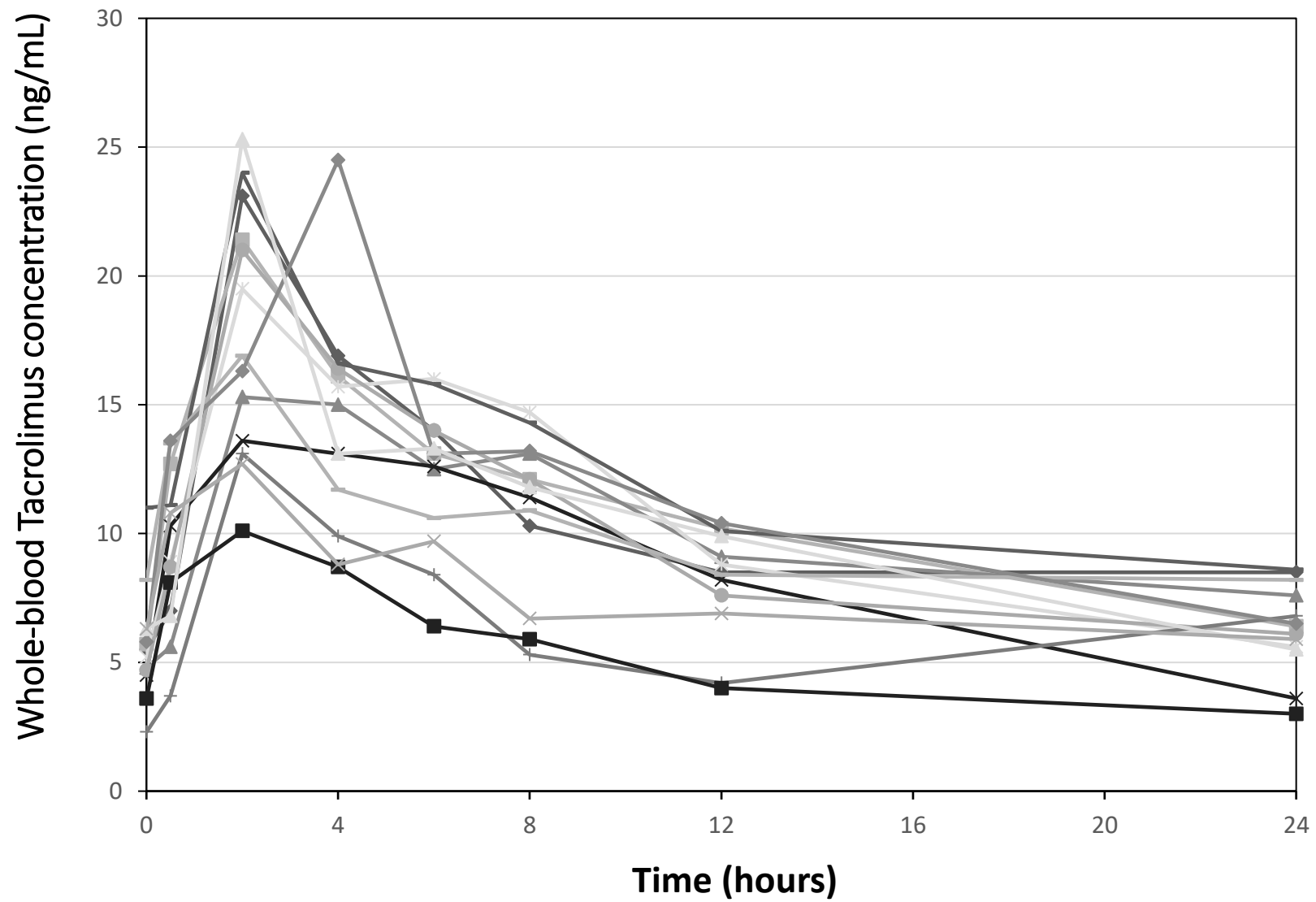

Supplement: S2 Fig — (PDF) [file pone.0225878.s002.pdf]

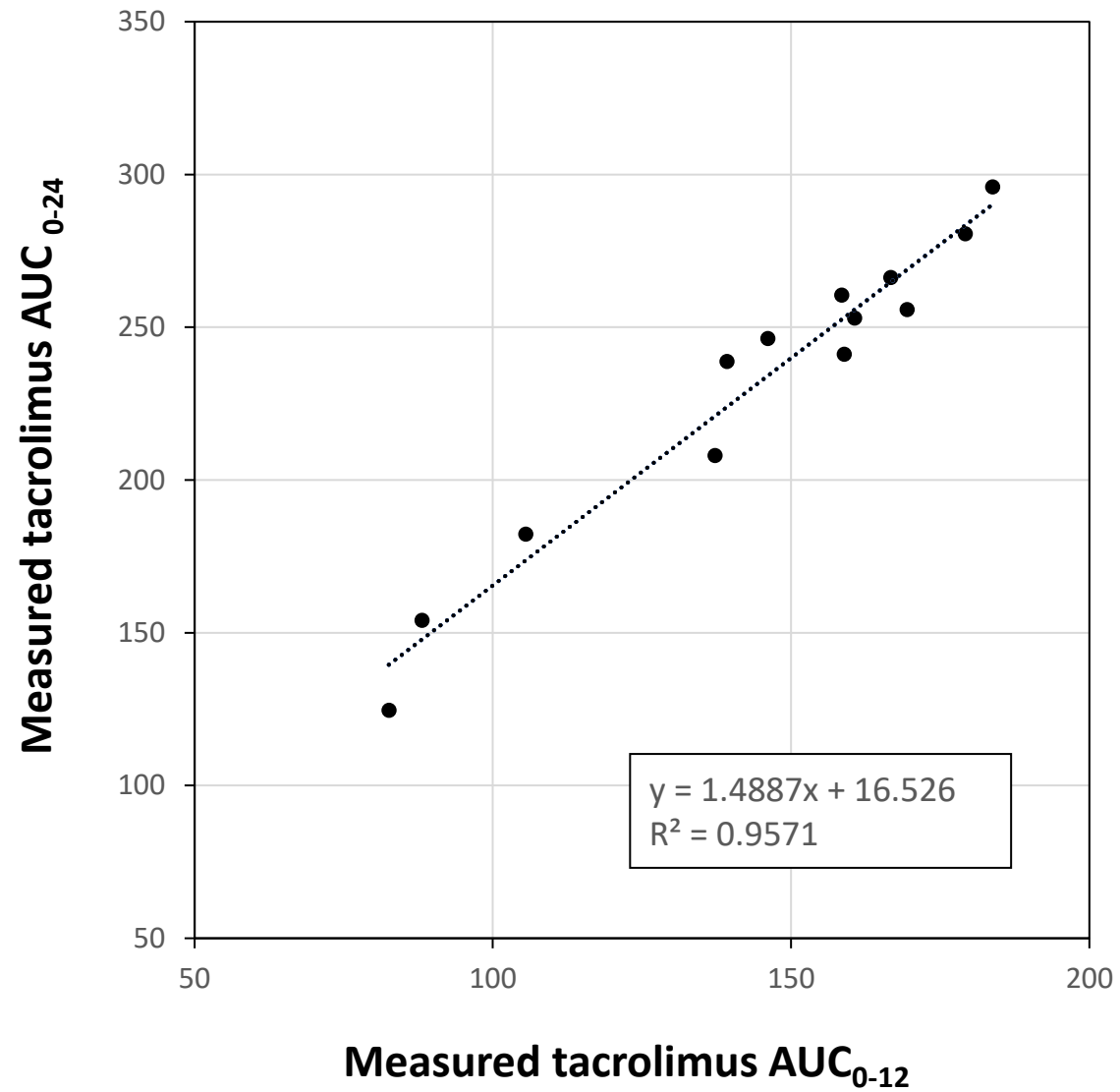

Supplement: S3 Fig — (PDF) [file pone.0225878.s003.pdf]
